# A Qualitative Scoping Review of Transgender and Gender Non-conforming People's Physical Healthcare Experiences and Needs

**DOI:** 10.3389/fpubh.2021.598455

**Published:** 2021-02-05

**Authors:** Michelle Teti, Steffany Kerr, L. A. Bauerband, Erica Koegler, Rebecca Graves

**Affiliations:** ^1^Department of Public Health, University of Missouri, Columbia, MO, United States; ^2^Department of Human Development and Family Science, University of Missouri, Columbia, MO, United States; ^3^Department of Health Sciences, University of Missouri, Columbia, MO, United States; ^4^School of Social Work, University of Missouri St. Louis, St. Louis, MO, United States; ^5^Health Sciences Library, University of Missouri, Columbia, MO, United States

**Keywords:** transgender, health care, health disparities, qualitative, scoping review transgender healthcare experiences and needs

## Abstract

Trans and gender non-conforming (TGNC) people experience poor health care and health outcomes. We conducted a qualitative scoping review of studies addressing TGNC people's experiences receiving physical health care to inform research and practice solutions. A systematic search resulted in 35 qualitative studies for analysis. Studies included 1,607 TGNC participants, ages 16–64 years. Analytic methods included mostly interviews and focus groups; the most common analysis strategy was theme analysis. Key themes in findings were patient challenges, needs, and strengths. Challenges dominated findings and could be summarized by lack of provider knowledge and sensitivity and financial and insurance barriers, which hurt TGNC people's health. Future qualitative research should explore the experiences of diverse and specific groups of TGNC people (youth, non-binary, racial/ethnic minority), include community-based methods, and theory development. Practice-wise, training for providers and skills and support for TGNC people to advocate to improve their health, are required.

Current research highlights the critical need to address health disparities and improve health outcomes among individuals who identify as transgender (1, 2). Transgender (trans) is an umbrella term for people whose gender identity, gender expression, or behavior does not conform to that typically associated with the sex to which they were assigned at birth (3). The term includes people who identify as gender non-conforming, a term that generally refers to people who do not adhere to the binary (e.g., male/female) concept of gender (3).

Trans and gender non-conforming (TGNC) people report many health disparities. For instance, discrimination and minority stress contribute to higher rates of poor mental health outcomes among TGNC people (4). These outcomes include, but are not limited to, depression (5–7), anxiety (5, 6), and suicide attempts (5, 7, 8). Trans women report much higher rates of HIV than the general population (9). Additionally, when compared to their cisgender counterparts, TGNC people report higher rates of disordered eating (5), smoking, obesity, and poor self-rated physical and mental health (10).

TGNC individuals are a vulnerable population due to the societal stigma associated with their identity (11), but they also experience unique hurdles to accessing quality healthcare. Despite the well-documented health disparities among TGNC individuals, research in this area is relatively new and only gained significance in the last decade (12). As understanding of the TGNC population and their health needs has increased, the community, itself, has changed to include more diverse identities and experience of gender (13). Many binary and non-binary TGNC individuals seek medical care to support physical changes that align with their gender identity (e.g., hormone therapy, surgery), increasing the importance of healthcare in the experience of being TGNC. However, given that transgender medicine is still a new area, many medical providers have received limited or no training in how to work with TGNC patients, and some hold biases associated with the binary approach to sex present in medicine (14). As a result of this, TGNC individuals may experience negative experiences in healthcare, not only as a result of societal stigma, but also due to competency issues among providers.

The most recent U.S. Transgender Survey indicated that TGNC people are also less likely to be insured than the general population and that notable numbers of trans people report negative health experiences (33%) or avoiding medical care (25%) (15). Relatedly, Edmiston et al. (1) reported that TGNC people are also less likely to access preventative health screenings. Lerner and Robles (16) identified discrimination from health care providers as a central cause of the underutilization of health care. Improving quality *health care experiences* for TGNC people, offers an opportunity to improve health outcomes, via enhanced care and decreased distress. We conducted a qualitative scoping review of studies addressing TGNC people's experiences receiving physical health care to implement more responsive TGNC care and improve trans health outcomes.

Prior reviews have begun to organize findings about TGNC health and health care. A recent review addressed TGNC persons' mental health care experiences (4). Cicero et al. (17) reviewed studies of transgender health care experiences to contextualize them within the Gender Affirmation Framework, categorizing health care experiences into social, medical, legal, and psychological barriers. Heng et al. (18) conducted a review of transgender people's general health care experiences—excluding specific health concerns like emergency care or HIV care.

The field of trans health is an emerging yet under researched area. Understanding current experiences is critical both for informing future healthcare research and adding to the literature needed to improve healthcare education. This analysis builds on prior reviews, to identify how to improve care and outcomes for TGNC people. First, the following analysis focuses exclusively on qualitative research and includes a quality assessment of the research using the consolidated criteria for reporting qualitative research (COREQ), a 32-item checklist for comprehensive reporting of qualitative studies (19). Qualitative research is well-suited to capture the nuances of patient experiences and needs (20). Assessing the quality of the research allows us to make recommendations for future qualitative directions and contributions in this field. Second, we also use theory-generating qualitative meta-synthesis methods (21). This approach provides a scoping summary of existing findings, and also cultivates generalizable theory pertaining to the research question. Third, we address *all* areas of physical health and do not exclude specific health concerns, such as HIV, to create a full picture of physical health that includes: challenges faced by TGNC people with health care providers and in health care systems.

## Materials and Methods

### Scoping Review

Our review was guided by five processes: (a) identify the research question driving the review, (b) identify relevant studies, (c) select studies, (d) extract data for selected categories, (e) analyze and synthesize findings, (f) and report data (21, 22). This study was approved by the primary author's institutional review board.

### Inclusion/Exclusion Criteria

To meet inclusion criteria, articles had to be peer reviewed, written in English and based on research studies that took place in the U.S. between January 1, 2008 and December 31, 2018. Other criteria included: the use of qualitative methods or inclusion of text, narratives, images, or artifacts as data; study samples with at least 50% trans men and/or trans women and/or gender non-conforming individuals who were represented in the findings; and the description of physical health care experiences with providers or physical health care as a major finding in the study. Methods articles, theoretical articles, presentations, and government and non-government reports were excluded.

Inclusion and exclusion criteria were created to meet our scoping review goals. We chose to limit our analysis to studies in the U.S., because personal, familial, legal, and social experiences of TGNC people differ greatly across cultures. These differences influence medical encounters for TGNC patients and our ability to integrate findings of those encounters meaningfully into U.S. findings. We limited the studies in this review to those that included at least 50% TGNC to ensure their representation in LGBT studies beyond the listing of the T in the acronym alone. Similarly, we limited our sample to studies with at least one theme addressing health care or provider experiences to ensure that these types of findings were adequately represented in the studies. We focused our review on physical health experiences because a recent review of mental health care experiences exists (4); and mental health often requires different approaches, interactions, and treatments than physical health experiences to be combined meaningfully in an analysis of physical health interactions.

### Search Procedures

The 5th author, in consultation with the first and third authors, created a search strategy. The base search strategy was constructed by an analysis of key terms in MeSH, and from relevant articles in MEDLINE, EMBASE, and CINAHL (see Table 1). The following databases were searched: EMBASE (Ovid), PubMed, CINAHL (EBSCOhost), Web of Sciences, CAB Direct, Gender Watch (ProQuest), and PsycINFO (EBSCOhost). All searches were run in May, 2019, and the base search was adapted to each database.

**Table 1:** Search strategy: CINAHL (EBSCO).

	**Search terms**	**Limits and search modes**
S6	S4 NOT S5	Limiters—English Language Search modes—Boolean/Phrase
S5	PT review OR review	Limiters—English Language Search modes—Boolean/Phrase
S4	S1 AND S2 AND S3	Search modes—Boolean/Phrase
S3	(MH “Qualitative Studies”) OR (MH “Action Research”) OR (MH “Ethnographic Research”) OR (MH “Ethnological Research”) OR (MH “Ethnonursing Research”) OR (MH “Grounded Theory”) OR (MH “Naturalistic Inquiry”) OR (MH “Phenomenological Research”) OR Qualitative OR Interview OR Interviews OR interviewed OR experience OR focus group OR themes	Limiters—English Language Search modes—Boolean/Phrase
S2	Health care OR healthcare OR Health needs OR health care needs OR healthcare needs OR health care barriers OR healthcare barriers OR health care facilitators OR healthcare facilitators OR Health care utilization OR healthcare utilization OR Health services needs OR “health services” OR Barriers to care OR Delivery of Health Care OR (MH “Health Resource Utilization”)	Limiters—English Language Search modes—Boolean/Phrase
S1	(MH “Transgender Persons”) OR Transgender OR Transgenders OR transgendered OR “trans men” OR “trans women” OR “trans person” OR “trans persons” OR “trans people” OR “trans patient” OR “gender minority” OR “gender non binary” OR “gender non-conforming” OR gender variant person OR gender variant persons OR genderqueer OR “non-binary person” OR “non-binary persons” OR “non-binary individual” OR “non-binary gender identity” OR transsexual OR transsexuals OR transexual OR transexuals OR transsexualism OR two spirit OR two spirited	Search modes—Boolean/Phrase

### Screening and Study Selection

**Figure 1:**
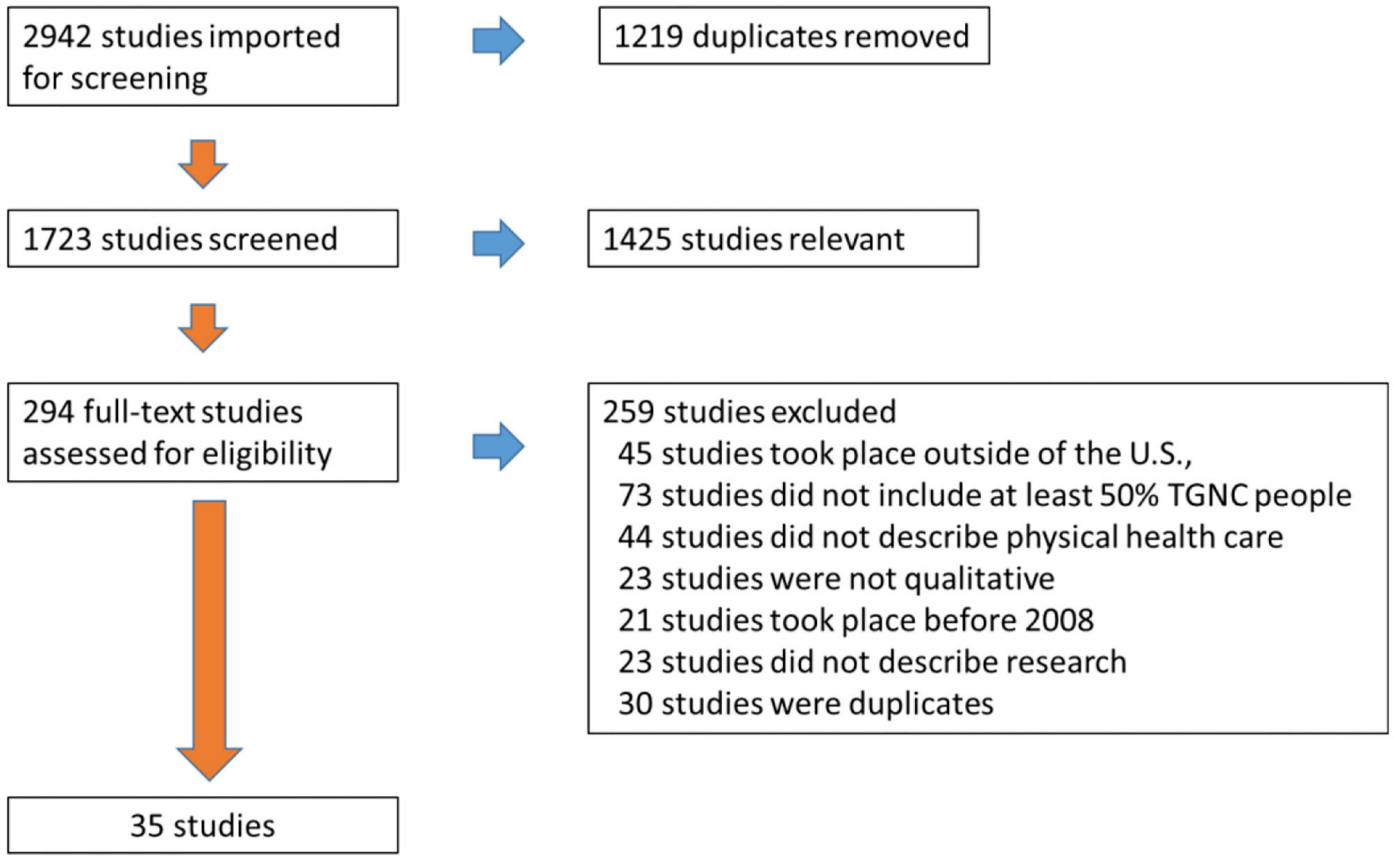
PRISMA.

The search identified a total of 2,942 citations. First, 1,219 duplicates were removed, leaving a total of 1,723 studies to be screened. The first four authors reviewed every title and abstract, performed the abstract review process, and reached consensus regarding articles relevant for further consideration (*n* = 294 articles). Two independent reviewers then read each full text article (*n* = 294) and used a screening form to verify inclusion criteria for each article. The reviewers then discussed each full text article that was being considered for inclusion. If there was discordance between the article and the inclusion criteria, the two reviewers presented the article to a third independent reviewer. Discussions ensued between all three reviewers until agreement occurred. At this point 259 articles were excluded for not meeting screening criteria: 45 studies took place outside of the U.S., 73 studies did not include at least 50% TGNC people, 44 studies did not describe physical health care, 23 studies were not qualitative, 21 studies took place before 2008, 23 studies did not describe research, and 30 studies were duplicates. As a result, 35 studies were retained for analysis. The screening and selection process was depicted using the PRISMA flowchart in Figure 1.

### Analysis

Analysis included collating important summary items to describe the depth and breadth of the articles, assessing article quality, and identifying key themes in the study results (21, 22). We extracted the following categories of data: inclusion criteria, sample size, key demographics (ethnicity, income, and gender), aim, recruitment settings, methods, analysis strategies, and findings. We extracted these categories of data to provide study overviews, support COREQ scoring, and facilitate theory development.

Quality of the articles was evaluated with the COREQ (19). COREQ items are grouped into three domains: research team and reflexivity, study design, and data analysis and reporting. Domain 1 addresses characteristics of the interviewer and the interviewer relationship with the participants with questions about interviewer credentials and how well the interviewer and participant know each other (eight items). Domain 2 addresses theoretical framework, participant selection, sample size, and data collection (15 items). Questions focus on where the data was collected and what the sample looks like. Domain 3 addresses data analysis and reporting (nine items) via explanations of themes and how participant quotes and input are integrated into the publication. We pilot-tested the COREQ with four articles, in which the first four authors reviewed their responses and discussed discrepancies until they were eliminated. At least two authors systematically reviewed each article by extracting all text related to the above categories, resolving any discrepancies, and producing a detailed data matrix that described basic components of the studies.

Data were then analyzed for the purpose of identifying generalizable themes and constructs that communicate transgender healthcare experiences within a working theoretical model (21). This process was described in detail along with the findings in the results section.

### Synthesis

We summarized and synthesized methodological aspects and demographics from each article in Tables 2, 3. We summarized and synthesized key elements from the COREQ in Tables 4–6.

**Table 2:** Description of study criteria and participants.

**References**	**Title**	**Inclusion criteria**	**Total # of participants**	**# Trans participants**	**Demographics**
Alpert et al. et al. (23)	What Lesbian, Gay, Bisexual, Transgender, Queer, and Intersex Patients Say Doctors Should Know and Do: A Qualitative Study	Aged 18+, LGBTQI or other related identities	25	25	Ethnicity: N/A. Age: 80% 25–35 years. Income: N/A. Gender: N/A.
Bith-Melander et al. (24)	Understanding Sociocultural and Psychological Factors Affecting Transgender People of Color in San Francisco	Not specified	Focus group-23, Interviews-20	43	Ethnicity: N/A. Age: N/A. Income: N/A. Gender: N/A
Chen et al. (25)	A qualitative analysis of transgender veterans' lived experiences	Aged 18+, prior service in U.S. Armed Forces, self-identified as transgender	201	201	Ethnicity: 86.5% white, 5% multiracial. Age: mean 49.9 years. Income: 39.3% $50,001. Gender: 67.7% transwomen, 18.4% split gender.
Chisolm-Straker et al. (26)	Transgender and Gender Non-conforming in Emergency Departments: A Qualitative Report of Patient Experiences	Self-identified as having a TGNC life experience, aged 18+, literate in English or Spanish, and needed or used an emergency department in the U.S.	240	240	Ethnicity: 82.5% white, 10.4% Latino/a. Age: 50.4% 25–35 years. Income: 16.3% < $11,000. Gender: 60.4% female, 21.7% male.
Cornelius and Whitaker-Brown (27)	African American Transgender Women's Individual, Family, and Organizational Relationships: Implications for Nurses.	Aged 18–35, self-identified as transgender females, African American, lived in North Carolina	15	15	Ethnicity: 100% African American. Age: 21–35 years. Income: 100% < $20,000. Gender: 100% transgender women.
Dewey (28)	Knowledge legitimacy: How trans-patient behavior supports and challenges current medical knowledge	Self-identified as trans	22	22	Ethnicity: 100% white. Age: Mean 48 years. Income: N/A. Gender: 91% born male but living full/part-time as female, 9% born intersex and living full time a women.
Dietert et al. (29)	Addressing the Needs of Transgender Military Veterans: Better Access and More Comprehensive Care	trans veterans, received healthcare services by the Veterans Health Administration	22	22	Ethnicity: 73% white, 9% unknown. Age: 31–71 years. Income: N/A. Gender: 73% transwoman, 23% transman.
Dunne et al. (30)	Interviews with Patients and Providers on Transgender and Gender Non-conforming Health Data Collection in the Electronic Health Record	Self-identified as TGNC, resident of Oregon, insured by Medicaid, received or currently receiving care from an Oregon community health clinic, providers who work in an Oregon community health clinic and have at least one TGNC patient.	7	7	Ethnicity: N/A. Age: N/A. Income: N/A. Gender: N/A
Dutton et al. (31)	Gynecologic Care of the Female-to-Male Transgender Man	Aged 18+, self-identified as transgender and of the female sex, lived within driving distance of the researcher	6	6	Ethnicity: 67% white, 17% black, 17% multiracial. Age: median 26.5 years. Income: 50% middle class. Gender: 83% male, 17% GNC.
Gridley et al. (32)	Youth and Caregiver Perspectives on Barriers to Gender-Affirming Health Care for Transgender Youth	Aged 14–22, self-identified transgender or caregiver of a transgender youth	15	15	Ethnicity: 67% white, 20% multiracial. Age youth: median 18 years. Income: N/A. Gender: 47% transmasculine, 33% other.
Hagen and Galupo (33)	Trans Individuals' Experiences of Gendered Language with Health Care Providers: Recommendations for Practitioners	Self-identified as trans, aged 18+	20	20	Ethnicity: 45% white, 20% black. Age: 20–64. Income: N/A. Gender: 30% trans male, 20% trans female, 20% female.
Hines et al. (34)	HIV Testing and Entry to Care Among Trans Women in Indiana	Aged 18+, living with HIV, self-identified as transgender, received services at an agency within the previous 12 months	18	18	Ethnicity: 56% African American, 22% white. Age: 21–60 years. Income: N/A. Gender: 89% transwomen, 11% male.
Hinrichs et al. (35)	Transgender and Gender Non-conforming Patient Experiences at a Family Medicine Clinic.	Aged 18 and older, English speaking, patients who had sought care at one urban clinic in relation to their TGNC identity or who had a diagnosis of gender dysphoria	23	23	Ethnicity: 77.3% white, 4.5% Asian/Pacific Islander, 4.5% American Indian/Alaskan, 4.5% Mexican Native. Age: 64% 25–44years. Income: N/A. Gender: 18.2% transgender male, 13.6% transgender woman.
Hoffkling et al. (36)	From erasure to opportunity: a qualitative study of the experiences of transgender men around pregnancy and recommendations for providers.	Aged 18+, self-identified as male before pregnancy, pregnant within the last 10 years, ability to write in English	10	10	Ethnicity: N/A. Age: 18+ years. Income: N/A. Gender: 100% transmen.
Kosenko et al. (37)	Transgender patient perceptions of stigma in health care contexts	Self-identified as transgender	152	152	Ethnicity: N/A. Age: mean 39.1 years. Income: N/A. Gender: 59% transexual, 14% gender queer.
Light et al. (38)	Transgender men who experienced pregnancy after female-to-male gender transitioning	Aged 18+, self-identified as male before pregnancy, pregnancy within the last 10 years, ability to write in English.	41	41	Ethnicity: 92% white, 3% Asian, 3% Asian and Black, 3% Native Hawaiian or other Pacific Islander. Age: 28 ± 6.8 years. Income: 49% $20,000–$59,000. Gender: 51% male, 24% transman.
Loza et al. (39)	A qualitative exploratory study on gender identity and the health risks and barriers to care for transgender women living in a U.S.–Mexico border city	Aged 18+, self-identified transgender woman, lived in El Paso, Texas	13	13	Ethnicity: 54% Latina/Mexican. Age: 20–late 60's. Income: N/A. Gender: 100% transwomen MTF.
Maragh-Bass et al. (40)	Is It Okay To Ask: Transgender Patient Perspectives on Sexual Orientation and Gender Identity Collection in Healthcare.	Aged 18+, English speaking, self-identified as transgender	101	101	Ethnicity: 58% white, 18% Hispanic. Age: mean 38 years. Income: N/A. Gender: 54% male, 46% female.
Melendez and Pinto (41)	HIV Prevention and Primary Care for Transgender Women in a Community-Based Clinic	Aged 18+, previously identified as a gender other than the gender they currently identified with, had received care at community-based clinic	20	20	Ethnicity: 80% Latina, 20% African American. Age: mean 30.7 years. Income: average monthly income $525. Gender: Transwomen.
McDowell et al. (6)	“It Can Promote an Existential Crisis:” Factors Influencing Pap Test Acceptability and Utilization Among Transmasculine Individuals	Ages 21–64, assigned female gender at birth, self-identified along the transmasculine spectrum, had a cervix	32	32	Ethnicity: 77% white. Age: mean 33 years. Income: median $10,000–$20,000. Gender: transmasculine.
Poteat et al. (42)	Managing uncertainty: A grounded theory of stigma in transgender health care encounters	Aged 18+, worked in the metropolitan area, provided medical care to at least one transgender patient in the preceding year	55	55	Ethnicity: 92% African American. Age: mean 36 years. Income: N/A. Gender: 55% transwomen, 45% transmen.
Puckett et al. (43)	Barriers to Gender-Affirming Care for Transgender and Gender Non-conforming Individuals	Aged 16+, lived in the U.S., self-identified as TGNC	201	201	Ethnicity: 78.9% white, 14.5% multiracial. Age: mean 28.4 years. Income: 45.3% < $10,000. Gender: 30.1% transmen, 23.4% transwomen.
Radix et al. (44)	Satisfaction and Healthcare Utilization of Transgender and Gender Non-Conforming Individuals in NYC: A Community-Based Participatory Study.	Self-identified as TGNC, aged 18+, English speaking, able to provide informed consent	46	46	Ethnicity: 35% African American, 24% Latino/a. Age: 18–64 years. Income: N/A. Gender: 45.7% transwomen, 17.4% transmen, 17.4% genderqueer.
Reisner et al. (45)	A mixed methods study of the sexual health needs of New England transmen who have sex with non-transgender men	Aged 18+, lived in New England, assigned female gender at birth, self-identified male or along the transmasculine spectrum, self-reported oral, anal, or vaginal sex with a non-transgender man in 12 months prior to study enrollment	16	16	Ethnicity: 87.5% white, 12.5% mixed race/ethnicity. Age: mean 32.5 years. Income: 63% $12,000+. Gender: 56.3% transgender.
Roller et al. (46)	Navigating the System: How Transgender Individuals Engage in Health Care Services	Self-identified as transgender, aged 21+, English speaking, have engaged in health care.	25	25	Ethnicity: 80% Caucasian, 8% African American. Age: majority 21–31 years. Income: N/A. Gender: 76% female, 20% male.
Rosentel et al. (47)	Transgender Veterans and the Veterans Health Administration: Exploring the Experiences of Transgender Veterans in the Veterans Affairs Healthcare System	Transgender veterans	11	11	Ethnicity: N/A. Age: N/A. Income: N/A. Gender: 82% women, 8% men.
Rowniak et al. (48)	Attitudes, beliefs, and barriers to PrEP among trans men	Self-identified trans men, aged 18+, English speaking	21	21	Ethnicity: 57% white, 14% African American, 14% Asian American. Age: Majority late 30's. Income: N/A. Gender: N/A
Samuels et al. (49)	“Sometimes You Feel Like the Freak Show:” A Qualitative Assessment of Emergency Care Experiences Among Transgender and Gender-Non-conforming Patients	Self-identified as trans, gender variant, gender queer, or intersex, aged 18+, visited a Rhode Island emergency department within the previous 5 years	32	30	Ethnicity: 78% white, 9% Black, 9% Asian/PI, 9% Native American. Age: 18+ years. Income: N/A. Gender: Male 46.9%, 43.8% transgender.
Sevelius et al. (50)	Barriers and facilitators to engagement and retention in care among transgender women living with human immunodeficiency virus	Aged 18+, living with HIV, able to provide informed consent, assigned male gender at birth and reporting gender identity as female, transgender female, or another trans identity indicating that they did not identify as male.	38	38	Ethnicity: 76% African American, 12% Latina. Age: 20–69 years. Income: 68% barely enough money to get by. Gender: 58 trans MTF.
Sevelius et al. (51)	“I am not a man:” Trans-specific barriers and facilitators to PrEP acceptability among transgender women	Aged 18+, sexually active within the past 3 months, assigned male gender at birth and reporting gender identity as female, transgender female, or another trans identity indicating that they did not identify as male.	30	30	Ethnicity: 36% multiracial, 26% white. Age: mean age 36 years. Income: N/A. Gender: 100% transwomen.
Singh et al. (52)	“I am my own gender:” Resilience strategies of trans youth	Self-identified as trans, aged 15–25	19	19	Ethnicity: 68% white, 16% multiracial. Age: mean 22 years. Income: 63% middle class. Gender: 58% transmen, 16% male.
Wagner et al. (53)	Health (Trans)gressions: Identity and Stigma Management in Trans* Healthcare Support Seeking	Not specified	17	17	Ethnicity: 94% white, 6% black. Age: 18–58 years. Income: N/A. Gender: transgender.
Wilkerson et al. (54)	Creating a culturally competent clinical environment for LGBT patients	Aged 18+, self-identified LGBT patient or LGBT healthcare provider	30	15	Ethnicity patient: 90% white, 10% Native American. Age: 18–64. Income: N/A. Gender patient: 30% male, 27% Trans ftm.
Wilson (55)	Access to HIV Care and Support Services for African American Transwomen Living with HIV	African American, transwomen, living with HIV	10	10	Ethnicity: 100% African American. Age: 28–55 years. Income: N/A. Gender: 100% transwomen.
Xavier et al. (56)	Transgender Health Care Access in Virginia: A Qualitative Study	Self-identified as trans	47	47	Ethnicity: 53% African American, 38% white. Age: N/A. Income: N/A. Gender: 68% transwomen, 32% trans men.

**Table 3:** Study recruitment, methods, and findings.

**References**	**Aim**	**Recruitment method**	**Analysis**	**Methods**	**Findings**
Alpert et al. (23)	Determine the competencies LGBTQI community members perceived physicians would need to deliver effective and accessible care	Transgender conference; transgender organizations; community contacts; social media; listservs	Thematic analysis	Focus groups	Competencies included a focus on patient autonomy and shared decision-making. Participants described the importance of avoiding gatekeeping or presenting obstacles to transition-related health care. Priorities included being comfortable with patients, avoiding assumptions/behaviors that reinforce stigma, increasing knowledge of sexual practices and transgender health, and working to decrease the effects of social determinants of health (particularly for marginalized subpopulations, including participants of color).
Bith-Melander et al. (24)	Provide information about the transgender community of color to help health care providers become more sensitive when serving this subpopulation.	Non-profit agencies	Not listed	Focus groups, interviews	Major barriers for transgender individuals' access to health care services included lack of health insurance, fear of not being able to see culturally competent and sensitive providers, lack of knowledge about available or free services, access to transportation, and access to healthcare. Participants identified the need for comprehensive health insurance that would cover transgender-related surgeries and follow-up (e.g., sensitive and continued gynecological services for FTM trans-gender men), mental health, and transitioning support and services to help transgender individuals come out and disclose their gender identities to family. HIV and mental health were also major concerns.
Chen et al. (25)	Address existing gaps in the literature to characterize both the challenges and strengths related to being a trans-vet.	Listserves; social media	Thematic analysis	Surveys	Recurring themes included health care access and providers, Veterans Health Administration (VA) and military experiences, discrimination, rejection vs. acceptance, concealment vs. authenticity, and the importance of community. Trans-vets also discussed feelings of personal strength, growth from adversity, and advocacy as important positive experiences. Findings demonstrated the centrality of military and VA experiences as unique aspects of transgender veteran identity.
Chisolm-Straker et al. (26)	Describe emergency department (ED) experiences of people with TGNC history and explore reasons why this population avoids ED, recommendations for care.	Community health centers that served lesbian, gay, bisexual, and TGNC-experienced persons; Facebook; a 2014 national conference on transgender health; word of mouth.	Thematic analysis	Retrospective, anonymous, written surveys (paper or web based)	Themes of self-efficacy and power inequity surfaced and exposed the tension between patients with TGNC experiences and clinicians who were perceived to lack training in their area, which resulted in negative patient experiences. When practitioners had specific training about this population, participants reported positive care experiences.
Cornelius et al. (27)	Gain an understanding of the process by which trans women develop relationships in society and in health care organizations.	Community contacts; participant referrals; social media	Content analysis	Interviews	The relationships of trans women were mostly negative at individual, family, and organizational levels.
Dewey (28)	Shed light on how trans-patients are made aware of, grasp, and incorporate knowledge into how they present their problems to medical staff.	Transgender organizations	Thematic analysis	Interviews	Trans-patients internalized views of knowledge legitimacy in ways that allowed them to make sense of their medical care. How trans-patients understand societal/medical views about them contributed to how they presented their needs to doctors, to ensure appropriate treatment. Some trans-patients, even if they utilized and supported medical knowledge to obtain their needs, engaged in forms of resistance and challenged medical knowledge.
Dietert et al. (29)	Investigate the experiences of transgender veterans with healthcare services provided by the VHA.	LGBT advocacy organizations; social media; participant referrals	Thematic analysis	Interviews	Although the VHA is working to address issues of inequality for transgender veterans, participants indicated that there are still problems with administration of care, proper training of staff and physicians, and availability of comprehensive services for the unique healthcare needs of transgender individuals.
Dunne et al. (30)	Explore how current electronic health record data collection practices affect the health and well-being of TGNC patients and how these practices can be modified to meet the needs of providers and patients.	Online	Thematic analysis	Interviews	Providers and trans patients want preferred pronouns, preferred name, and gender identifier in a forward-facing display. Patients expressed the need for a broader range of gender and birth-assigned sex identifiers. Patients felt that they should not have to divulge birth-assigned sex unless they wanted to. Physicians and clinicians felt this was crucial information to document for primary care. Patients and providers had differing opinions on who should be tasked with collecting TGNC health information.
Dutton et al. (31)	Inform healthcare literature, mid-wives, and women's health care nurse practitioners regarding the gynecologic needs of the transgender male community.	Listserves; community agencies	Phenomenology	Interviews	Trans men had a wide variety of coping mechanisms for receiving gynecologic care, which were often dependent on whether or not they revealed their gender identity and biologic sex incongruence. Interviewees struggled with whether to mark female because they had a vagina or male because they appeared masculine when sitting in the waiting room and gender-identified as male. Language was a barrier to receiving care. Pronoun and name usage by the health care provider and staff appeared to be a second barrier.
Gridley et al. (32)	Identify barriers to accessing gender-affirming health care; solicit recommendations for overcoming these barriers.		Thematic analysis	Interviews	Barriers were: few accessible trained providers for TGNC youth, lack of consistent protocols, inconsistent pronoun use, uncoordinated care and gatekeeping, limited/delayed access to blockers and hormones, insurance exclusions.
Hagen and Galupo (33)	Allow trans individuals agency to tell their own experiences and discover the ways patient/provider communication could be improved to improve health care	Personal contacts within different trans communities; trans -focused online listservs	Thematic analysis	Interviews	Changes need to be made by health care providers and their offices to make them more inclusive, affirming, and respectful of trans identities. Recommendations are separated into three primary categories: medical forms, interpersonal communication, and whole person care.
Hines et al. (34)	Describe the circumstances influencing HIV testing and entry to care among transgender women in Indiana.	Transgender support agencies	Content analysis	Interview and survey	Participants identified three circumstances under which they received HIV testing and diagnosis: for most it was during a routine screening, a few initiated HIV testing on their own, and a few initiated HIV testing at the advice or urging of another. Most participants linked to HIV care within 3 months of the initial diagnosis while other participants experienced delayed entry to care. Factors that motivated participants to seek care after a delay were psychosocial support, education or information support, provider persistence, seeing others die from HIV, and substance abuse treatment.
Hinrichs et al. (35)	To learn from TGNC patients about their care at a family medicine clinic; to find out how primary care clinics can improve care for TGNC patients.	Electronic medical records of patients at one urban clinic in Minneapolis	Grounded Theory	Focus groups	Four main themes emerged: (1) shared negative experiences with health care, (2) the need for sensitive and inclusive primary care, (3) defining TGNC-sensitive care, and (4) the challenges of mainstreaming TGNC-competent care into primary care settings
Hoffkling et al. (36)	To understand the needs of transgender men who had given birth.	Online	Grounded Theory	Interviews	There were a range of experiences and needs of patients. Findings revealed broad diversity in the experiences, circumstances, and degrees of empowerment of men who are pregnant and give birth. Adequate care was rarely reported by participants.
Kosenko et al. (37)	Explore transgender patients' experiences with health care with a focus on their negative experiences.	Online links and mail to LGBT organizations across the U.S.	Content analysis	Surveys	Negative interactions with various health professionals included gender insensitivity, displays of discomfort, denial of services, substandard care, verbal abuse, and forced care.
Light et al. (38)	Explore the experiences of transgender men; contribute to the knowledge base of fertility, conception, pregnancy experience, and birth outcomes among transgender men.	LGBT health centers; transgender community groups; social media	Grounded Theory	Surveys	Themes emerged as views on pregnancy in the context of family structure, the relationship between gender dysphoria and pregnancy, and feelings of isolation. Participants desired more information regarding fertility options and access to reproductive health care providers who respect, support, and understand their gender identity.
Loza et al. (39)	Examine the health-related risks and barriers to care for transgender women in a U.S. Mexico border city; fill gaps in the literature in relation to transgender health among a Latina population.	Networking with community gatekeepers and LGBT-friendly venues	Phenomenology	Interviews	3 main themes include self-acceptance of trans identity, acceptance of trans identity within social networks, and health risks (e.g., body modifications and barriers to health care). Findings reveal phases of self-acceptance of trans identity, a high level of health risks, scarce health services resources, and low levels of acceptance from family, friends, and partners.
Maragh-Bass et al. (40)	To understand the views of transgender patients on routine sexual orientation/gender identity (SO/GI) collection in healthcare.	Urban academic medical center market research firm	Inductive coding	Surveys	Many patients reported that medical relevance to their chief complaint and an LGBT-friendly environment would increase willingness to disclose their SO/GI. Patients also reported a need for educating providers in LGBT health prior to implementing routine SO/GI collection. Personal, environmental, contextual, and political factors impacted how participants felt about being asked and responding to SO/GI information.
Melendez et al. (41)	Examine how a community-based clinic that offers free or low-cost care addresses the health care needs of transwomen.	Community-based medical clinic	Content analysis	Interviews	Factors reported to be effective for HIV prevention and primary care included access to health care in settings not dedicated to serving transgender and/or gay communities, a friendly atmosphere and staff sensitivity, and holistic care including hormone therapy.
McDowell et al. (6)	Examine the factors influencing Pap test utilization among transmasculine individuals to inform evidence-based interventions to promote regular cervical cancer screening.	Fenway Health Center; local community-based organizations serving transgender individuals; social media; local Pride event	Grounded Theory	Interviews	A wide range of experiences with Pap testing was reported. Many participants who had experienced extreme obstacles to screening in the past stated that these barriers were diminished or eliminated when they went to a more respectful provider.
Poteat et al. (42)	Explore how stigma and discrimination functions in health care encounters between transgender patients and medical providers.	LGBT health center; transgender support groups; trans organizations; referral by community advisory board and participants	Grounded Theory	Interviews	Themes included feelings about transgender identities, feelings about hormone therapy, learning about transgender health, clinical interactions with transgender patients, and interactions with colleagues. Uncertainty emerged as a recurrent theme throughout categories, which challenged the traditional clinical relationship. Interpersonal stigma can serve to reinforce the traditional provider patient power relationship. Some respondents internalized stigma and wrestled with self-hatred or projected negative attitudes toward other trans people. Most had learned to anticipate discrimination, which led some to limit their geographic, employment, and health care options.
Puckett et al. (43)	Examine rates of pursuing/desiring to pursue different forms of gender-affirming healthcare (i.e., hormone therapy, top surgery, bottom surgery, puberty blockers) and barriers encountered for each	Social media (e.g., Facebook, Twitter, Tumblr); community organizations that served transgender community.	Thematic analysis	Surveys (open ended)	Barriers to care were financial, insurance and employment, availability of care, bias and stigma in medical field, interpersonal barrier (e.g., no social support), emotions/worries making it difficult to approach providers, concerns with quality, lack of information, non-trans related medical issues, aging and timing of care.
Radix et al. (44)	Assess the needs of TGNC individuals in New York City with a focus on HIV/STIs, Pap smears, colorectal screening, routine care, and hormonal use, to improve service provision.	Community spaces and venues where TGNC individuals congregate (e.g., community-based organizations and special-events); direct referrals from health care professionals serving transgender communities	Comprehensive process analysis and grounded theory	Focus groups	Barriers to health care utilization included medical providers' inadequate knowledge of transgender health issues and lack of cultural competency working with TGNC people. Participants noted difficulty accessing culturally-competent surgeons, difficulty navigating the legal system for name change or gender marker or accessing assistance, and feeling invisible and/or misrepresented in the public health arena. To minimize isolation, participants suggested holding “Trans Health Nights,” creating welcoming physical spaces, producing a TGNC newsletter, and community building through social events.
Reisner et al. (45)	Gain a deeper understanding of the sexual health concerns and needs of transmen, including but not limited to HIV and STD risk; explore the influence of gender dynamics in sexual encounters with non-transgender men.	Fenway Health Institute	Content analysis	Interviews	Important aspects of HIV and STD prevention intervention design and delivery were: integrated sexual health information “by and for” transgender men into other healthcare services, involving peer support, addressing mood and psychological well-being (e.g., depression and anxiety), Internet-delivered information for transmen and their sexual partners, and training for health care providers.
Roller et al. (46)	Conduct theoretical framework that depicts the process by which transgender individuals (TI) engage in healthcare	Transgender websites; listservs; sites of online support groups	Grounded Theory	Interviews	The central phenomenon of how TIs engage in health care was the core process of navigating the system. Sub-processes included needing to move forward, doing due diligence, finding loopholes, and making it work.
Rosentel et al. (47)	Explore transgender veterans' experiences accessing and utilizing transition-related healthcare through the VA healthcare system.	Event for transgender veterans	Qualitative inquiry methodological perspective and experience-centered approach	Interviews	Themes impacting the accessibility and quality of care transgender veterans receive through the VA include long delays in receiving care; needing to travel to receive care; lack of patient knowledge regarding the coverage of transition-related care; insensitivity, harassment, and violence among providers; a general lack of knowledge about transgender patients and care among providers
Rowniak et al. (48)	Examine the attitudes and knowledge of trans men regarding pre-exposure prophylaxis (PrEP) for HIV.	LGBT Community Center; 2 health clinics serving trans patients; sex club with special nights for trans men; websites	Thematic analysis	Focus groups	Themes included the range of information about PrEP and possible side effects, the economic realities for trans men, finding a trans-competent provider, trans male sexuality, the importance of contraception, and condom use. A lack of access to PrEP was noted.
Samuels et al. (49)	Understand trans patient care barriers and experiences at every point of contact in an emergency department, from triage through clinician encounters and diagnostic testing, to generate suggestions for improvement.	Listservs; magazines; LGBT organizations; LGBT businesses; local Pride event	Grounded Theory	Focus groups and surveys	Themes included system-based barriers to care, overt discrimination, lack of clinician competence in trans care, and emotional trauma incurred from the ED experience. Privacy, communication, and provider competency were priority areas for improvement.
Sevelius et al. (50)	Examine the barriers and facilitators unique to transwomen in order to elucidate disparities in engagement and retention in HIV care; provide insight for those wishing to understand and mitigate the forces that result in disproportionately poor health outcomes.	Community-based agencies that serve transgender women	Template analysis	Interviews and focus groups	Challenges for adhering to HIV care and treatment included avoidance of healthcare due to stigma and past negative experiences, prioritization of hormone therapy, and concerns about adverse interactions between antiretroviral treatment for HIV and hormone therapy. Receiving culturally competent, transgender-sensitive healthcare was a powerful facilitator of health care empowerment.
Sevelius et al. (51)	Fill a gap in the literature by exploring trans-specific facilitators and barriers to PrEP acceptability among a sample of urban trans women at risk for HIV acquisition in San Francisco.	Community-based organizations and service sites	Concept analysis	Interviews and focus groups	Knowledge of PrEP was low; interest was relatively high once participants were informed. Due to past negative healthcare experiences, ability to obtain PrEP from a trans-competent provider was cited as essential to PrEP uptake and adherence. Participants noted that PrEP could address situations in which trans women experience reduced power to negotiate safer sex, including sex work. Trans-specific barriers included lack of trans-inclusive marketing of PrEP, prioritization of hormone use, and medical mistrust due to transphobia.
Singh et al. (52)	Examine barriers and resilience in navigating societal discrimination (e.g., trans-prejudice, adultism) to help counselors and other helping professionals understand how to further support the development of trans youth clients' resilience.	Social media (list-serves, Facebook, Twitter)	Phenomenology	Interviews	5 themes of resilience were: ability to self-define and theorize one's gender, proactive agency and access to supportive educational systems, connection to a trans-affirming community, reframing of mental health challenges, and navigation of relationships with family and friends.6 major threats to participants' resilience were: experiences of adultism, health care access challenges, emotional and social isolation, employment discrimination, limited access to financial resources, and gender policing.
Wagner et al. (53)	Explore stigma through interviews with trans individuals to help transcend the framing that trans people are helpless, passive, and in need of rescuing	Advocacy agencies, LGBTQ Resource Centers, personal connections, transgender studies listserv	Open coding	Interviews	Trans identity is fraught with anxiety surrounding perceptions, responses, and validation of that identity. Participants had considerable fear when it came to seeking healthcare support. Many situated their identity as necessary to acknowledge in healthcare contexts, but recognized that acknowledgment could lead to discrimination.
Wilkerson et al. (54)	Fill gaps in understanding of how health care providers and patients differ in their perceptions of a culturally competent health care environment and how health care administrators and educators can support the implementation of culturally competent LGBT health care.	LGBT-friendly businesses and community-based organizations; listservs; an LGBT event	Thematic analysis	Focus groups	Findings identified a culturally competent clinical environment consisted of structural components (e.g., décor, patient flow), systemic components (e.g., mission statements, policies, forms), and interpersonal components (e.g., trusting provider-patient relationship). Strategies to create a more culturally competent clinical environment were identified
Wilson (55)	Fill a gap in the literature about structural and individual-level barriers to care among African American HIV+ transwomen outside San Francisco in Alameda County, California	Local community-based organization	Thematic analysis	Interviews	Themes of gender stigma, peer, and institutional distrust provide insight into African American transwomen's barriers to HIV care and support services. Access to care was impacted by whether organizations offered gender-related care, the geography of organizations related to safe transportation and location, confidentiality and trust of peers and organizations, and trauma.
Xavier et al. (56)	Identify factors associated with risk of HIV infection and the social determinants of health status among transgender people in Virginia; examine how trans people access routine medical, mental health, trans related, and HIV services in Virginia.	Direct outreach by trusted community leaders	Descriptive coding	Focus groups	Victimization associated with social stigmatization played a dominant role in participants' lives, manifested by discrimination; violence; and health care provider insensitivity, hostility, and ignorance of transgender health. Access to transgender-related medical services that would allow participants to pass in their chosen genders was their highest medical priority. Faced with barriers to access, hormonal self-medication was common, and silicone injections were reported by both MTF and FTM participants. Due to economic vulnerability, sex work was reported as a source of income by both MTF and FTMs. MTFs expressed concern over confidentiality of HIV testing and additional discrimination if testing positive. FTMs expressed difficulty accessing gynecological care due to their masculine gender identities and expressions.

**Table 4:** COREQ domain 1: research team and reflexivity.

**References**	**Identity**	**Credentials**	**Occupation**	**Gender**	**Experience**	**Relationship**	**Participant knowledge**	**Interviewer characteristics**
Alpert et al. (23)	1	1	1	1	1	1	1	1
Bith-Melander et al. (24)	0	1	1	0	0	0	0	0
Chen et al. (25)	na	1	na	na	na	na	na	na
Chisolm-Straker et al. (26)	na	1	0	na	na	na	na	na
Cornelius and Whitaker-Brown (27)	0	1	1	0	0	0	0	0
Dewey (28)	1	0	0	0	0	0	0	0
Dietert et al. (29)	0	0	0	0	0	0	0	0
Dunne et al. (30)	1	1	1	0	1	0	0	0
Dutton et al. (31)	1	1	1	0	1	0	0	0
Gridley et al. (32)	0	1	0	0	0	0	0	0
Hagen and Galupo (33)	1	0	0	0	0	0	0	0
Hines et al. (34)	1	1	1	1	1	0	0	1
Hinrichs et al. (35)	1	0	0	0	0	0	0	0
Hoffkling et al. (36)	1	0	0	0	0	0	0	0
Kosenko et al. (37)	na	0	1	na	na	na	na	na
Light et al. (38)	na	1	0	na	na	na	na	na
Loza et al. (39)	1	0	0	0	0	0	0	0
Maragh-Bass et al. (40)	na	1	1	na	na	na	na	na
Melendez and Pinto (41)	0	0	1	0	0	0	0	0
McDowell et al. (6)	1	1	1	1	1	0	0	0
Poteat et al. (42)	1	1	1	1	1	1	1	1
Puckett et al. (43)	na	0	0	na	na	na	na	na
Radix et al. (44)	1	1	0	0	1	0	1	0
Reisner et al. (45)	0	1	0	0	0	0	0	0
Roller et al. (46)	0	1	1	0	1	0	0	0
Rosentel et al. (47)	1	1	0	0	0	0	0	0
Rowniak et al. (48)	1	1	1	1	0	0	0	1
Samuels et al. (49)	1	1	0	0	0	0	0	0
Sevelius et al. (50)	1	0	0	1	1	0	1	1
Sevelius et al. (51)	1	0	1	0	0	0	0	0
Singh et al. (52)	1	1	1	na	1	na	0	na
Wagner et al. (53)	0	0	0	0	0	0	0	0
Wilkerson et al. (54)	0	0	0	0	0	0	0	0
Wilson (55)	1	1	1	0	1	0	0	0
Xavier et al. (56)	1	0	0	1	1	1	1	1
NA totals	6	0	1	7	6	7	6	7
No totals	9	14	18	7	17	25	7	22
Yes totals	20	21	16	21	12	3	22	6

**Table 5:** COREQ domain 2: study design.

**References**	**Method orientation**	**Sample**	**Method of approach**	**Sample size**	**Non-participation**	**Setting**	**Non-participants**	**Description of sample**	**Interview guide**	**Repeat interviews**	**Audio recording**	**Field notes**	**Duration**	**Data saturation**	**Transcripts returned**
Alpert et al. (23)	1	1	1	1	1	1	1	1	1	1	1	1	1	1	1
Bith-Melander et al. (24)	1	1	1	1	0	1	1	1	1	1	1	na	1	0	0
Chen et al. (25)	1	1	1	1	1	1	na	1	1	0	na	na	1	0	0
Chisolm-Straker et al. (26)	1	1	0	1	0	1	0	1	na	0	na	na	na	1	0
Cornelius and Whitaker-Brown (27)	1	1	1	1	1	0	0	1	1	1	1	0	1	1	0
Dewey (28)	1	1	1	1	0	0	0	1	na	0	1	1	0	0	0
Dietert et al. (29)	1	1	1	1	0	na	0	1	1	0	0	0	0	0	0
Dunne et al. (30)	1	1	1	1	0	1	1	1	1	0	0	1	1	0	0
Dutton et al. (31)	1	1	1	1	0	1	1	1	1	0	1	1	1	0	0
Gridley et al. (32)	1	1	0	1	0	1	0	1	1	0	1	0	1	1	0
Hagen and Galupo (33)	1	1	1	1	0	1	0	1	1	1	1	0	1	0	1
Hines et al. (34)	1	1	1	1	1	1	0	1	1	na	1	0	1	0	0
Hinrichs et al. (35)	1	1	1	1	0	1	1	1	1	1	1	0	1	0	0
Hoffkling et al. (36)	1	1	1	1	1	1	0	1	1	0	1	0	0	1	0
Kosenko et al. (37)	1	1	na	1	0	1	na	1	1	0	na	na	na	1	na
Light et al. (38)	1	1	1	1	0	na	na	1	1	0	0	na	0	0	0
Loza et al. (39)	1	1	1	1	0	1	0	1	1	0	1	0	1	0	0
Maragh-Bass et al. (40)	1	1	na	1	1	1	na	1	na	na	na	na	na	0	na
Melendez and Pinto (41)	1	1	1	1	0	1	1	1	1	0	1	0	1	0	0
McDowell et al. (6)	1	1	1	1	0	1	1	1	1	0	1	0	1	1	0
Poteat et al. (42)	1	1	1	1	0	1	0	1	na	1	1	1	1	1	0
Puckett et al. (43)	1	1	1	1	1	1	0	1	na	na	na	na	na	0	na
Radix et al. (44)	1	1	1	1	0	0	0	1	1	0	0	0	1	0	0
Reisner et al. (45)	1	1	1	1	0	1	0	1	1	0	1	0	1	1	0
Roller et al. (46)	1	1	1	1	0	1	0	1	1	0	1	1	0	1	0
Rosentel et al. (47)	1	1	1	1	0	1	0	1	1	0	1	0	1	1	0
Rowniak et al. (48)	1	1	1	1	0	1	1	1	1	0	1	1	1	0	0
Samuels et al. (49)	1	1	1	1	0	0	1	1	1	0	1	0	1	0	0
Sevelius et al. (50)	1	1	0	1	0	0	0	1	1	na	1	0	1	0	0
Sevelius et al. (51)	1	1	0	1	0	0	0	1	1	0	1	1	1	0	0
Singh et al. (52)	1	1	1	1	1	1	0	1	1	0	1	1	1	0	na
Wagner et al. (53)	1	1	1	1	0	1	0	1	1	0	1	0	1	1	0
Wilkerson et al. (54)	1	1	0	1	0	1	0	1	1	0	1	0	1	0	0
Wilson (55)	1	1	0	1	0	1	0	1	1	0	1	1	1	0	0
Xavier et al. (56)	1	1	0	1	1	1	0	1	1	1	1	1	0	0	0
NA totals	0	0	2	0	0	2	4	0	5	4	5	7	4	0	4
No totals	0	0	7	0	26	6	22	0	0	24	4	17	6	23	29
Yes totals	35	35	26	35	9	27	9	35	30	7	26	11	25	12	2

**Table 6:** COREQ domain 3: analysis and findings.

**References**	**Coders**	**Coding tree**	**Derivation of themes**	**Software**	**Participant checking**	**Quotations**	**Data and findings consistent**	**Major themes**
Alpert et al. (23)	1	1	1	1	1	1	1	1
Bith-Melander et al. (24)	0	0	0	1	0	1	1	1
Chen et al. (25)	1	1	1	0	1	1	1	1
Chisolm-Straker et al. (26)	1	1	na	1	1	na	1	1
Cornelius and Whitaker-Brown (27)	1	0	1	0	0	1	1	1
Dewey (28)	1	1	1	0	0	1	1	1
Dietert et al. (29)	0	0	1	0	0	1	1	0
Dunne et al. (30)	0	0	1	0	1	1	1	1
Dutton et al. (31)	1	0	1	0	0	1	1	1
Gridley et al. (32)	0	1	1	1	0	1	1	1
Hagen and Galupo (33)	1	1	1	1	0	1	1	1
Hines et al. (34)	1	1	1	0	0	1	1	1
Hinrichs et al. (35)	1	1	1	1	0	1	1	1
Hoffkling et al. (36)	1	0	0	0	0	0	0	0
Kosenko et al. (37)	na	0	1	na	na	na	na	na
Light et al. (38)	0	1	1	0	0	1	1	1
Loza et al. (39)	0	0	1	1	1	1	1	1
Maragh-Bass et al. (40)	na	1	1	na	na	na	na	na
Melendez and Pinto (41)	1	1	1	0	0	1	1	1
McDowell et al. (6)	0	1	1	1	0	1	1	1
Poteat et al. (42)	1	1	1	1	1	1	1	1
Puckett et al. (43)	1	1	1	0	0	1	1	1
Radix et al. (44)	0	0	1	0	0	1	1	1
Reisner et al. (45)	0	0	1	1	0	1	1	1
Roller et al. (46)	1	0	1	0	1	1	1	1
Rosentel et al. (47)	1	1	1	1	0	1	1	1
Rowniak et al. (48)	1	1	1	1	0	1	1	1
Samuels et al. (49)	1	1	1	1	0	1	1	1
Sevelius et al. (50)	1	0	1	1	0	1	1	1
Sevelius et al. (51)	1	1	1	1	0	1	1	1
Singh et al. (52)	0	na	1	0	1	1	1	1
Wagner et al. (53)	1	0	1	0	0	1	1	1
Wilkerson et al. (54)	1	1	1	1	0	1	1	1
Wilson (55)	1	0	1	1	0	1	1	1
Xavier et al. (56)	1	0	1	1	0	1	1	1
NA totals	0	1	1	1	0	0	0	0
No totals	10	15	1	14	27	2	0	1
Yes totals	25	19	33	20	8	33	35	34

## Results

### Study Descriptions

Table 2 describes inclusion criteria, sample size, and participant demographics. The majority of studies addressed trans patient experiences with general care [e.g., (23, 28, 42, 45)], but six studies focused specifically on HIV care [e.g., (34, 41, 48)], four on obstetrics and gynecological care [e.g., (36, 38)], three on veteran health care clinics (25, 29, 47) and two on emergency room care (26, 49).

All studies took place in the United States (U.S.) and included transgender participants. Authors defined trans in different ways by most commonly including participants who identified as “trans” [e.g., (24, 30, 34, 46)], “trans or gender non-conforming” [e.g., (35, 43, 44)] or as having a different gender identity compared to the birth identity [e.g., (41, 51)]. One study included LGBTQ participants, of which 50% identified as trans (54) and one study included two intersex participants (49). For all other studies, TGNC comprised 100% of the study population. Just over half of the studies included participants who were 18 or older (*n* = 19). The most common age inclusion criteria was very broad or included participants aged 16, 18, 21, or 25 years and older without upward limits [e.g., (38–40, 48)]. Eligibility criteria for a few studies included age ranges, and two of those articles limited eligibility to youth under age 25 (32, 52). Study sample sizes ranged from 6 to 201 participants.

The actual demographic make-up of the study participants was as follows:

- Gender: This review represents 1,624 participants and 1,607 TGNC participants. Most studies included trans men and trans women; six studies included trans women only [e.g., (37, 55)] and six included trans men only [e.g., (36, 57)].
- Age: Following inclusion criteria, participants in these studies ranged from 16 to 64 years.
- Ethnicity: Three studies included 100% African American or mostly African American samples (over 90%) (27, 42, 55) and two studies had all or mostly White participants (over 90%) (38, 53). One study had mostly Latinx participants (41). The majority of studies that included race/ethnicity had a mix of ethnicities although over 10 studies included over 70% White samples. Few other races/ethnicities were strongly represented besides White, Black, or Latinx.

### Study Methods Summary

Table 3 outlines study aims, recruitment settings, methods, analysis strategies, and key findings. The majority of study aims focused on exploring trans people needs, experiences, use of, or engagement in care. Other study aims centered specifically on barriers to seeking care [e.g., (32, 43)], understanding cultural competency and stigma [e.g., (23, 33, 49)], or how to improve care [e.g., (24, 38)]. Several studies highlighted trans people's resilience, facilitators, or strengths related to care [e.g., (25, 52)].

Almost all studies used a mix of recruitment methods. The most common settings were health or community centers that might serve or attract trans participants [e.g., (23, 28, 51)], social media or the web more generally [e.g., (25, 27, 52)], personal contacts [e.g., (42, 56)] or at trans-specific events [e.g., (49, 54, 57)].

The most common method that researchers used to study trans experiences were interviews [e.g., (29, 39, 53)], open-ended surveys [e.g., (37)], or focus groups [e.g., (23, 48, 56)]. A few studies used interviews and focus groups together. The most common analytic strategies were variations of thematic or content analysis [e.g., (26, 34, 45)]. A few authors cited phenomenology (31, 39, 52) or Grounded Theory [e.g., (35, 36, 46)].

### Themes in Findings

Findings were varied, given varied study aims, populations, and methods (see Table 3). We conducted a theme analysis of study findings to identify themes (21). An initial round of coding identified three major themes: health care challenges, health care needs, and TGNC resources and strengths. Challenges were the largest category of findings and included a lack of provider knowledge and or sensitivity (e.g., poor training, lack of competency, hostile treatment environments, stigma, discrimination, harassment) and financial and insurance barriers (poverty, lack of affordable care for basic health and gender affirming health changes, lack of access to care, inadequate insurance). Needs included improved care and knowledge from providers, peer support, patient autonomy, and patient-informed practices. TGNC patient strengths were persistence as a self-advocate, resilience amid adversity in life and in healthcare, and willingness to grow from adversity.

To better understand the largest category of themes, challenging experiences within the health system, we conducted a second round of coding in which findings of each research article were analyzed for mentions of major challenges—“Lack of provider knowledge and sensitivity” and “Financial/insurance barriers” and affiliated mentions of consequences (e.g., “Trickle-down effects). Code frequencies and overlaps were used to draw connections between challenges and their effects. Figure two articulates a theoretical model to conceptualize the larger impact of TGNC people's healthcare experiences (21).

### Trickle-Down Effects: A Model for Conceptualizing Larger Impact

#### Lack of Provider Knowledge and Sensitivity

Many qualitative findings captured the detriments of lack of provider knowledge [e.g., (31, 36, 46, 53)]. Primary data illustrated that providers lacking adequate training or awareness to provide gender-affirming healthcare resulted in adverse healthcare experiences for TGNC patients. For instance, practitioners often provided unnecessary probing, questioning, or physical examinations as a result of their lack of knowledge. Practitioners were often misinformed about a potential lack of congruence between gender identity and biological anatomy, which resulted in treatment that was overly invasive or not medically necessary. Conversely, TGNC individuals frequently reported the lack of medically necessary treatment as a result of a lack of education on behalf of the medical provider. For instance, healthcare providers would often neglect to perform medically necessary cancer screenings, STI education and care, or in extreme cases, emergency medical care for illness or injury. Findings also indicated frequent harassment, misgendering, and hostile responses, ranging from humiliating the TGNC patient, refusing appropriate privacy, or asking the patient to leave the facility.

Codes nested under the theme of “Lack of provider knowledge and sensitivity” were most commonly associated with the trickle-down effects of “Long-term lack of healthcare” and “Avoidable injury, sickness, or death.” Primary qualitative data revealed that the trickle-down effects of lacking provider knowledge resulted in sustained, long-term lack of necessary physical healthcare, either as a result of fear-based avoidance, or blatant refusal of medically necessary, preventative healthcare [e.g., (23, 24, 27, 28, 35, 37, 40, 42, 43, 48, 50, 51, 56)]. Moreover, a lack of appropriate medical care often resulted in reported avoidable injury, illness, or in some circumstances, possible death of the TGNC individual. For example, qualitative evidence highlighted a range of severity, from infections resulting from emergency care practitioners ignoring symptoms or refusing care [e.g., (26, 37, 54)], cancer emerging from a lack of preventative screening [e.g., (31, 45)], or lack of life-saving treatments administered [e.g., (50, 51)]. Evidence suggested that benign ignorance about TGNC healthcare needs can have severe, life threatening consequences for TGNC patients.

#### Financial and Insurance Barriers

While there was some overlap with the sub-theme of “Lack of provider knowledge,” this sub-theme was distinct in that it highlighted more systemic financial and procedural barriers faced by TGNC individuals within the healthcare system. From a societal standpoint, gender affirming healthcare such as hormone therapy and surgical procedures are often not covered by insurance, and TGNC individuals are also significantly less likely to have medical coverage (23, 42, 44, 56). This leaves cost burden on the TGNC patient, resulting in debt and forces TGNC people to choose between allocating limited financial resources to either standard, preventative healthcare, or gender affirming care. Additionally, financial burden also hinders the ability to attend to prerequisite healthcare necessary for eligibility for gender affirming care. Primary qualitative data consistently described TGNC individuals that were unable to afford preliminary healthcare appointments required for receiving hormone therapy (43, 53), which sometimes led TGNC patients to seek and obtain gender affirming treatments on the black market, and/or through self-administered methods.

As a result, the trickle down effects of financial, insurance, or procedural barriers were associated with a lack of long-term healthcare and avoidable injury, illness, or possible death [e.g., (23, 26, 34, 38, 41, 45, 54, 56)]. In addition to the health risks of forced resource allocation choice or black market procedures, primary qualitative data repeatedly highlighted that TGNC individuals were more likely to turn to sex work in order to afford healthcare.

### Study Quality Summary

Tables 4–6 outline the results of the COREQ. Domain one addressed research team and reflexivity with eight sub-domains. Subdomains were evaluated aggregately to understand the patterns of meeting criteria among qualitative studies of TGNC people's experience in health care. Thus, a perfect score for each sub-domain was 35 because there are 35 studies and each study received a point for addressing a domain. Average scores for each sub-domain in domain one were outlined in Table 4. The average score across all domain one sub-domain scores was 15.1. Several sub-domains in domain one were not relevant for scoring. This was true for online studies with no human interviewer for whom to consider for reflexivity issues (e.g., interviewer identity and characteristics, gender, experience/training, relationship with or knowledge of participants). Domain scores ranged from three studies to 21 studies receiving a check point for the domains (see Table 4). Studies (20 or more out of 35) most commonly identified interviewer identity; interviewer gender; and participant knowledge of the interviewer, as their reasons for doing the research. Studies also frequently identified the credentials of the primary author of the study. Few studies have determined if the interviewer established a relationship with participants prior to the study (only three studies) and reported characteristics about the interviewer (only six studies), such as potential interviewer biases and assumptions.

Domain two addressed study design with 15 sub-domains. Summary scores for each sub-domain are in Table 5. The average score across all domain two scores was 21.6. Domain scores ranged from two to 35 studies receiving a check point for the domains. Nearly all (35/35) of the studies identified study methodological orientation or theory, sampling strategy, sample size, and sample description. Many studies (e.g., 20 or more) reported the study duration and setting, method of recruiting participants, description of an interview guide, and whether or not interviews were recorded. Few studies described who refused to participate or dropped out of the study (nine studies), who was present besides researchers or participants in the interviews (nine studies), if repeat interviews were carried out (seven studies) or if transcripts were returned to participants for corrections or comments (two studies).

Domain three addressed analysis and findings with nine sub-domains. Average scores for each sub-domain are in Table 6. The average score across all sub-domains was 26.3. Domain three scores ranged from eight to 35 studies receiving check points for the domains. Thirty or more studies described how they derived themes from the data, presented results that were consistent with their analyses, described major and minor themes, and provided participant quotations. Only eight studies commented on allowing the participants the opportunity to give feedback on the findings.

## Discussion

This scoping review summarized elements of studies addressing TGNC people's experiences of receiving physical health care and identified directions for theory, research, and practice to explain and address TGNC people's health care experiences. Reviews to date cover TGNC people's experiences of mental health care and limited aspects of physical health care. This review builds on and expands those findings.

### Stigma and Discrimination

White and Fontenot (4) found that although participants did report welcoming mental health care environments, most also experienced stigma and discrimination, which was worse for racial/ethnic minority TGNC persons. Our findings indicated that TGNC people face similar challenges in physical health care, and in addition to the stigma, report that providers lack competency, education, and ability to give patients referrals for more complex physical needs like surgeries. Other recent reviews on aspects of TGNC people's physical health care experiences included a focus on discrimination and stigma at the provider, office, and medical system level (4, 18). Moreover, our findings highlighted that the consequences of adverse health system experiences are pervasive across the life span, and throughout all domains of biological, psychological, and social health. Indirect consequences resulting include lack of ongoing healthcare and life-long avoidance and/or fear of the health system, sub-standard health care that does not meet the health needs of the individual, and in the worst case, avoidable injury or death.

TGNC people also reported lack of access to adequate care, which reflects discrimination at the structural level. For example, problems attaining employment, which could relate to employment discrimination, can hurt health care options. Lack of insurance coverage for services that TGNC people need—such as reassignment surgeries—also reflects a larger system bias against understanding these processes as necessary.

### Resilience Amid Discrimination

Despite challenges, our findings also add an awareness of patient identified areas of strengths. Studies in this review identified the importance of positive influences like peer support, patient autonomy, and patient-informed practices. Additionally, TGNC patients are resilient, and willing to self-advocate if given opportunities and skills. Such resiliency, if respected and built upon by providers, could help TGNC people navigate the health care system more successfully.

### Provider Impact and Trickle-Down Consequences of TGNC Care

**Figure 2:**
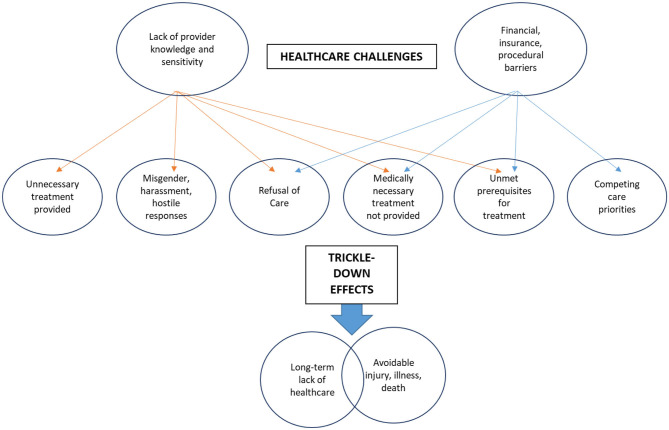
Trickle-down effects: a model for conceptualizing larger impact.

The theoretical model produced (Figure 2) highlights extensive thematic analysis for the purpose of presenting a conceptual model to aid healthcare practitioners in comprehending the severe consequences of adverse healthcare experiences for TGNC individuals. This model highlights the life-long health consequences of such experiences. By highlighting the trickle-down challenges of TGNC patients, health practitioners have the capacity to better understand the long-term effects of their medical decisions and practices. In addition, health practitioners can better conceptualize the impetus for obtaining appropriate gender-affirming training and understanding the health needs of TGNC patients.

### Limitations of Existing TGNC Healthcare Data

This review also highlighted several notable issues in the existing body of qualitative work on TGNC people's physical health care experiences. On one hand, the amount of information generated by qualitative studies was rich, detailed, and revealing. On the other hand, study samples were limited demographically. There were inconsistencies in how each research team defined trans people which made it challenging to compare and collate research results (58). Also, as trans research grows, and different groups under the trans umbrella become more visible, it is important to acknowledge the heterogeneity of trans people and their needs. Recent research, for example, notes that non-gender conforming groups report worse health outcomes than those who identify as trans male or female (59–61). Given that one of the strengths of qualitative research is to explore in depth the experiences of specific groups of people, studies differentiated by varied trans identities may be more informative than studies that have a broad sample of all TGNC identities.

Relatedly, studies addressed the experiences of a wide range of ages. It was common for studies to include samples of “18 and older.” Different life phases bring different health challenges, however. Research points toward distinct disparities and health concerns for very young (62) and aging trans people (63), for example. Future studies may be able to generate even more useable results if they focus on specific life experiences, like youth, adolescence, mid-life or older adulthood. Only three studies focused on the experiences of African American TGNC people, despite research that intersections of identity, such as ethnicity and gender, matter, for an assessment of health care experiences and outcomes (64).

Similarly, most of the studies in this review covered health generally, a very broad topic. Specific studies were limited to explorations of HIV or obstetric health care or the delivery of veteran or emergency room services. Given the range of health disparities experienced by trans people, an understanding of broader experiences of physical care are needed.

Several studies used online surveys, which resulted in larger samples. In other areas of research on sensitive topics, like sexual behavior, for example, online surveys or mechanisms of answering sensitive questions elicit higher reports of sensitive risk behaviors (65). Limitations exist as well, such as the inability to explore issues in depth with an interviewer. Online surveys are necessary for the broad recruitment of TGNC samples but may miss the nuances of regional experiences. Additional research should investigate the pros and cons of online surveys verses local interviews.

The most common methods used in the studies were individual or group interviews. Although these methods are valuable, there may be a place for additional methods, like ethnography or visual methods in this field, to supplement existing research with observations of doctor visits to assist in the description of the health care experience. These methods may add depth to existing studies.

Relatedly, the review identified only one community based participatory research (CBPR) study (66). CBPR is a partnership approach to research in which researchers and communities work together to define research problems, determine how to study them, and translate results into action. Given the success of CBPR in conducting collaborative research and disseminating collaborative results with marginalized communities (66, 67), CBPR qualitative studies may be a helpful addition to this field of work and may further drive research questions and answer to solutions. The studies in this review predominantly used theme analysis, which was a beneficial tool in identifying patterns in data. As the field of TGNC health grows, moving beyond identification of patterns to more complex forms of analysis, like theory generation, will help move the field forward (20).

### Study Limitations

Our review was subject to several limitations. Although systematic, it was possible that we excluded articles despite our library search. The COREQ is a valuable instrument to assess the quality of qualitative work (19). In the context of this analysis, however, limitations did exist. Domain 1, regarding the research team, assumes an in-person interaction, not an online survey. Given the rising popularity of on-line qualitative instruments, challenges arise when using the COREQ. The gender item under reflexivity was stated as a binary (e.g., was the researcher male or female) which was not always relevant to our studies. Some of the other topics were uncommon in our studies all-together. For example, the presence of non-participants in an interview setting and conducting repeating interviews (domain 2) were not entirely clear and were frequently not reported. The first three items in domain 3 (e.g., were major themes presented clearly?) are widely open to interpretation, unlike many of the other specific elements of the instrument. In addition, we limited the review to U.S. based studies. The review does not capture the experience of TGNC in developing and/or countries outside of the U.S.

Those limitations notwithstanding, this was the first study, to our knowledge to evaluate qualitative trans studies using the COREQ, which offered important patterns for attention in future research. The least commonly reported items in domain one were the characteristics of the interviewer and the interviewer relationship with the participant. Excluding limiting measures listed above, least commonly reported items in domain two were data saturation and information about the people who refused participation in the study. In domain three, participant checking was least reported.

### Future Research Directions

Our analysis also identifies important areas for future study and action regarding the physical health care experiences of TGNC people. The experiences of diverse TGNC including gender, age, ethnic and other diverse aspects of identity—and diverse health care experiences, and resiliency, are in need of further study. These strategies are particularly important for qualitative research that explore subpopulations in great detail. Methods beyond interview and focus group alone, such as ethnography, visual methods, or participatory approaches may add to current qualitative findings. Going beyond capturing themes to building theory will be critical. Measures beyond the COREQ, to capture the growing field of online interviewing, will assist with building the field.

### Future Practice Directions

Our findings reveal the need for change in the physical health care system, as the health consequences for transgender individuals are severe and perpetuate life-long health disparities. For the purpose of informing health professionals about the severity of stigma, discrimination, and challenges within the health environment for TGNC individuals, findings from our analysis were used to develop a theoretical model of trickle-down effects of adverse experiences (21). This model synthesizes salient themes and connects challenges identified within this qualitative scoping review to latent trickle-down effects of adverse experiences (see Figure 2). This model was intended to accompany findings reported in this study, while also translating research findings directly to practice.

## Author Contributions

MT conceptualized the article, led the analysis, and writing of the article. SK took part in article review and wrote significant parts of the discussion. LB took part in article review and helped with the introduction. EK helped with article review and article editing. RG led the search for articles. All authors contributed to the article and approved the submitted version.

## Conflict of Interest

The authors declare that the research was conducted in the absence of any commercial or financial relationships that could be construed as a potential conflict of interest.

## References

[1] Edmiston EK Donald CA Rose Sattler A Klint Peebles J Ehrenfeld JM Eckstrand KL. Preventative health services for transgender patients: a systemic review. Transgender Health. (2016) 1:216–30. 10.1089/trgh.2016.0019 28861536 PMC5367473

[2] Safer JD Coleman E Feldman J Garofalo R Hembree W Radix A Barriers to healthcare for transgender individuals. Curr Opin Endocrinol Diabetes Obesity. (2016) 23:168–71. 10.1097/MED.0000000000000227 PMC4802845 26910276

[3] American Pschological Association Transgender People, Gender Identity and Gender Expression. (2019). Available online at: https://www.apa.org/topics/lgbt/transgender

[4] White BP Fontenot HB. Transgender and non-conforming persons' mental healthcare experiences: an integrative review. Arch Psychiatric Nurs. (2019) 33:203–10. 10.1016/j.apnu.2019.01.005 30927991

[5] Lefevor GT Boyd-Rogers CC Sprague BM Janis RA. Health disparities between genderqueer, transgender, and cisgender individuals: an extension of minority stress theory. J Counsel Psychol. (2019) 66:385–95. 10.1037/cou0000339 30896208

[6] McDowell MJ Hughto JMW Reisner SL. Risk and protective factors for mental health morbidity in a community sample of female-to-male trans-masculine adults. BMC Psychiatry. (2019) 19:16. 10.1186/s12888-018-2008-0 30626372 PMC6327526

[7] Tebbe EA Moradi B. Suicide risk in trans populations: an application of minority stress theory. J Counsel Psychol. (2016) 63:520–33. 10.1037/cou0000152 27089059

[8] Johns MM Lowry R Andrzejewski J Barrios LC Demissie Z McManus T. Transgender identity and experiences of violence victimization, substance use, suicide risk, and sexual risk behaviors among high school students - 19 states and large urban school districts, 2017. Morbid Mortality Weekly Rep. (2019) 68:67–71. 10.15585/mmwr.mm6803a3 30677012 PMC6348759

[9] Becasen JS Denard CL Mullins MM Higa DH Sipe TA. Estimating the prevalence of HIV and sexual behaviors among the US transgender population: a systematic review and meta-analysis, 2006-2017. Am J Public Health. (2018) 109:e1–8. 10.2105/AJPH.2018.304727 30496000 PMC6301428

[10] Streed CG McCarthy EP Hass JS Association between gender minority stress and self-reported physical and mental health in the United States. JAMA Internal Med. (2017) 177:1210–1. 10.1001/jamainternmed.2017.1460 28558100 PMC5818796

[11] Hendricks ML Testa RJ A conceptual framework for clinical work with transgender and gender nonconforming clients: an adaptation of the Minority Stress Model. Professional Psychol Res Pract. (2012) 43:460–7. 10.1037/a0029597

[12] Institute of Medicine The Health of Lesbian, Gay, Bisexual, and Transgender People: Building a Foundation for Better Understanding. (2011). Washington, DC Available online at: http://www.nationalacademies.org/hmd/Reports/2011/The-Health-of-Lesbian-Gay-Bisexual-and-Transgender-People.aspx 22013611

[13] Motmans J Nieder TO Bouman WP. Transforming the paradigm of nonbinary transgender health: a field in transition. Int J Transgend. (2019) 20:119–25. 10.1080/15532739.2019.1640514 32999599 PMC6830970

[14] Korpaisarn S Safer JD. Gaps in transgender medical education among healthcare providers: a major barrier to care for transgender persons. Rev Endocr Metab Disord. (2018) 19:271–5. 10.1007/s11154-018-9452-5 29922962

[15] James SE Herman JL Rankin S Keisling M Mottet L Anafi M The Report of the 2015 U.S.Transgender Survey. (2016). Available online at: https://transequality.org/sites/default/files/docs/usts/USTS-Full-Report-Dec17.pdf

[16] Lerner JE Robles G Perceived barriers and facilitators to health care utilization in the United States for transgender people: a review of recent literature. J Health Care Poor Underserved. (2017) 28:127–52. 10.1353/hpu.2017.0014 28238993

[17] Cicero EC Reisner SL Silva SG Merwin EI Humphreys JC. Health care experiences of transgender adults: an integrated mixed research literature review. Adv Nurs Sci. (2019) 42:123–38. 10.1097/ANS.0000000000000256 30839332 PMC6502664

[18] Heng A Heal C Banks J Preston R Transgender people's experiences and perspectives about general healthcare: a systematic review. Int J Transgenderism. (2018) 19:359–78. 10.1080/15532739.2018.1502711

[19] Tong A Sainsbury P Craig J. Consolidated criteria for reporting qualitative research (COREQ): a 32-item checklist for interviews and focus groups. Int J Qual Health Care. (2007) 19:349–57. 10.1093/intqhc/mzm042 17872937

[20] Corbin J Strauss A Basics of Qualitative Research. 3rd ed. Los Angeles, CA: Sage Publications (2008).

[21] Fingfeld-Connett D A Guide to Qualitative Meta-Synthesis. New York, NY: Routledge (2018).

[22] Arksey H O'Malley L Scoping studies: towards a methodological framework. Int J Soc Res Methodol. (2005) 8:19–32. 10.1080/1364557032000119616

[23] Alpert AB CichoskiKelly EM Fox AD. What lesbian, gay, bisexual, transgender, queer, and intersex patients say doctors should know and do: a qualitative study. J Homosexual. (2017) 64:1368–89. 10.1080/00918369.2017.1321376 28481724 PMC6947913

[24] Bith-Melander P Sheoran B Sheth L Bermudez C Drone J Wood W. Understanding sociocultural and psychological factors affecting transgender people of color in San Francisco. J Assoc Nurses AIDS Care. (2010) 21:207–20. 10.1016/j.jana.2010.01.008 20416495

[25] Chen JA Granato H Shipherd JC Simpson T Lehavot K A qualitative analysis of transgender veterans' lived experiences. Psychol Sexual Orientation Gender Diversity. (2017) 4:63–74. 10.1037/sgd0000217

[26] Chisolm-Straker M Jardine L Bennouna C Morency-Brassard N Coy L Egemba MO. Transgender and gender nonconforming in emergency departments: a qualitative report of patient experiences. Transgender Health. (2017) 2:8–16. 10.1089/trgh.2016.0026 28861544 PMC5367487

[27] Cornelius JB Whitaker-Brown CD. African American transgender women's individual, family, and organizational relationships: implications for nurses. Clin Nurs Res. (2017) 26:318–36. 10.1177/1054773815627152 26810439

[28] Dewey JM. Knowledge legitimacy: how trans-patient behavior supports and challenges current medical knowledge. Qualitative Health Res. (2008) 18:1345–55. 10.1177/1049732308324247 18832767

[29] Dietert M Dentice D Keig Z. Addressing the needs of transgender military veterans: better access and more comprehensive care. Transgender Health. (2017) 2:35–44. 10.1089/trgh.2016.0040 28861546 PMC5436371

[30] Dunne MJ Raynor LA Cottrell EK Pinnock WJA. Interviews with patients and providers on transgender and gender nonconforming health data collection in the electronic health record. Transgender Health. (2017) 2:1–7. 10.1089/trgh.2016.0041 28861543 PMC5367482

[31] Dutton L Koenig K Fennie K. Gynecologic care of the female-to-male transgender man. J Midwifery Womens Health. (2008) 53:331–7. 10.1016/j.jmwh.2008.02.003 18586186 PMC4902153

[32] Gridley SJ Crouch JM Evans Y Eng W Antoon E Lyapustina M. Youth and caregiver perspectives on barriers to gender-affirming health care for transgender youth. J Adolesc Health. (2016) 59:254–61. 10.1016/j.jadohealth.2016.03.017 27235374

[33] Hagen DB Galupo MP Trans^*^ individuals' experiences of gendered language with health care providers: recommendations for practitioners. Int J Transgenderism. (2014) 15:16–34. 10.1080/15532739.2014.890560

[34] Hines DD Draucker CB Habermann B. HIV testing and entry to care among trans women in Indiana. J Assoc Nurses AIDS Care. (2017) 28:723–36. 10.1016/j.jana.2017.05.003 28652131 PMC5572502

[35] Hinrichs A Link C Seaquist L Ehlinger P Aldrin S Pratt R Transgender and gender nonconforming patient experiences at a family medicine clinic. Acad Med. (2018) 93:76–81. 10.1097/ACM.0000000000001837 28767493

[36] Hoffkling A Obedin-Maliver J Sevelius J. From erasure to opportunity: a qualitative study of the experiences of transgender men around pregnancy and recommendations for providers. Pregnancy Childbirth. (2017) 17:332. 10.1186/s12884-017-1491-5 29143629 PMC5688401

[37] Kosenko K Rintamaki L Raney S Maness K. Transgender patient perceptions of stigma in health care contexts. Med Care. (2013) 51:819–22. 10.1097/MLR.0b013e31829fa90d 23929399

[38] Light AD Obedin-Maliver J Sevelius JM Kerns JL. Transgender men who experienced pregnancy after female-to-male gender transitioning. Obstetr Gynecol. (2014) 124:1120–7. 10.1097/AOG.0000000000000540 25415163

[39] Loza O Beltran O Mangadu T A qualitative exploratory study on gender identity and the health risks and barriers to care for transgender women living in a U.S.–Mexico border city. Int J Transgenderism. (2017) 18:104–18. 10.1080/15532739.2016.1255868

[40] Maragh-Bass AC Torain M Adler R Ranjit A Schneider E Shields RY. Is it okay to ask: transgender patient perspectives on sexual orientation and gender identity collection in healthcare. Acad Emerg Med. (2017) 24:655–67. 10.1111/acem.13182 28235242

[41] Melendez RM Pinto RM. HIV prevention and primary care for transgender women in a community-based clinic. J Assoc Nurses AIDS Care. (2009) 20:387–97. 10.1016/j.jana.2009.06.002 19732697 PMC3534725

[42] Poteat T German D Kerrigan D. Managing uncertainty: a grounded theory of stigma in transgender health care encounters. Soc Sci Med. (2013) 84:22–9. 10.1016/j.socscimed.2013.02.019 23517700

[43] Puckett JA Cleary P Rossman K Newcomb ME Mustanski B. Barriers to gender-affirming care for transgender and gender nonconforming individuals. Sex Res Soc Policy. (2018) 15:48–59. 10.1007/s13178-017-0295-8 29527241 PMC5842950

[44] Radix AE Lelutiu-Weinberger C Gamarel KE. Satisfaction and healthcare utilization of transgender and gender non-conforming individuals in NYC: a community-based participatory study. LGBT Health. (2014) 1:302–8. 10.1089/lgbt.2013.0042 26789858

[45] Reisner SL Perkovich B Mimiaga MJ. A mixed methods study of the sexual health needs of New England transmen who have sex with nontransgender men. AIDS Patient Care STDS. (2010) 24:501–13. 10.1089/apc.2010.0059 20666586 PMC2958438

[46] Roller CG Sedlak C Draucker CB. Navigating the system: how transgender individuals engage in health care services. J Nurs Scholarship. (2015) 47:417–24. 10.1111/jnu.12160 26243380

[47] Rosentel K Hill BJ Lu C Barnett JT. Transgender veterans and the veterans health administration: exploring the experiences of transgender veterans in the veterans affairs healthcare system. Transgender Health. (2016) 1:108–16. 10.1089/trgh.2016.0006 29159302 PMC5685269

[48] Rowniak S Ong-Flaherty C Selix N Kowell N. Attitudes, beliefs, and barriers to PrEP among trans men. AIDS Educ Prev. (2017) 29:302–14. 10.1521/aeap.2017.29.4.302 28825860

[49] Samuels EA Tape C Garber N Bowman S Choo EK. “Sometimes you feel like the freak show”: a qualitative assessment of emergency care experiences among transgender and gender-nonconforming patients. Ann Emerg Med. (2018) 71:170–82.e171. 10.1016/j.annemergmed.2017.05.002 28712604

[50] Sevelius JM Patouhas E Keatley JG Johnson MO. Barriers and facilitators to engagement and retention in care among transgender women living with human immunodeficiency virus. Ann Behav Med. (2014) 47:5–16. 10.1007/s12160-013-9565-8 24317955 PMC3925767

[51] Sevelius JM Keatley J Calma N Arnold E. 'I am not a man': Trans-specific barriers and facilitators to PrEP acceptability among transgender women. Global Public Health. (2016) 11:1060–75. 10.1080/17441692.2016.1154085 26963756 PMC10204128

[52] Singh AA Meng SE Hansen AW 'I am my own gender': resilience strategies of trans youth. J Counsel Dev. (2014) 92:208–18. 10.1002/j.1556-6676.2014.00150.x

[53] Wagner PE Kunkel A Asbury MB Soto F Health (Trans)gressions: identity and stigma management in trans^*^ healthcare support seeking. Women Lang. (2016) 39:49–74.

[54] Wilkerson J Rybicki M Barber S Cheryl A Smolenski DJ Creating a culturally competent clinical environment for LGBT patients. J Gay Lesbian Soc Serv. (2011) 23:376–94. 10.1080/10538720.2011.589254

[55] Wilson EC Arayasiriku S Johnson K. Access to HIV care and support services for African American transwomen living with HIV. Int J Transgenderism. (2014) 14:182–95. 10.1080/15532739.2014.890090 24817835 PMC4012687

[56] Xavier J Bradford J Hendricks ML Safford L McKee R Martin E Transgender health care access in virginia: a qualitative study. Int J Transgenderism. (2013) 14:13–7. 10.1080/15532739.2013.689513

[57] Peitzmeier SM Agenor M Bernstein IM McDowell M Alizaga NM Reisner SL. “It can promote an existential crisis”: factors influencing pap test acceptability and utilization among transmasculine individuals. Qualitative Health Res. (2017) 27:2138–49. 10.1177/1049732317725513 28836483

[58] Reisner SL Deutsch MB Bhasin S Bockting W Brown GR Feldman J. Advancing methods for US transgender health research. Curr Opin Endocrinol Diabetes Obesity. (2016) 23:198–207. 10.1097/MED.0000000000000229 26845331 PMC4916925

[59] Aparicio-García ME Díaz-Ramiro EM Rubio-Valdehita S López-Núñez MI García-Nieto I. Health and well-being of cisgender, transgender and non-binary young people. Int J Environ Res Public Health. (2018) 15:2133. 10.3390/ijerph15102133 30274141 PMC6209926

[60] Olson-Kennedy J Cohen-Kettenis PT Kreukels BP Meyer-Bahlburg HF Garofalo R Meyer W. Research priorities for gender nonconforming/transgender youth: gender identity development and biopsychosocial outcomes. Curr Opin Endocrinol Diabetes Obesity. (2016) 23:172–9. 10.1097/MED.0000000000000236 26825472 PMC4807860

[61] Rider GN McMorris BJ Gower AL Coleman E Eisenberg ME. Health and care utilization of transgender and gender nonconforming youth: a population-based study. Pediatrics. (2018) 141:e20171683. 10.1542/peds.2017-1683 29437861 PMC5847087

[62] Connolly MD Zervos MJ Barone CJ 2nd Johnson CC Joseph CL. The mental health of transgender youth: advances in understanding. J Adolesc Health. (2016) 59:489–95. 10.1016/j.jadohealth.2016.06.012 27544457

[63] Fredriksen-Goldsen KI Cook-Daniels L Kim HJ Erosheva EA Emlet CA Hoy-Ellis CP. Physical and mental health of transgender older adults: an at-risk and underserved population. Gerontologist. (2014) 54:488–500. 10.1093/geront/gnt021 23535500 PMC4013724

[64] Bowleg L. The problem with the phrase women and minorities: intersectionality - an important theoretical framework for public health. Am J Public Health. (2012) 102:1267–73. 10.2105/AJPH.2012.300750 22594719 PMC3477987

[65] Macalino GE Celentano DD Latkin C Strathdee SA Vlahov D. Risk behaviors by audio computer-assisted self-interviews among HIV-seropositive and HIV-seronegative injection drug users. AIDS Educ Prev. (2002) 14:367–78. 10.1521/aeap.14.6.367.24075 12413183

[66] Israel BA Coombe CM Cheezum RR Schulz AJ McGranaghan RJ Lichtenstein R. Community-based participatory research: a capacity-building approach for policy advocacy aimed at eliminating health disparities. Am J Public Health. (2010) 100:2094–102. 10.2105/AJPH.2009.170506 20864728 PMC2951933

[67] Olshansky E Sacco D Braxter B Dodge P Hughes E Ondeck M Participatory action research to understand and reduce health disparities. Nursing Outlook. (2005) 53:121–6. 10.1016/j.outlook.2005.03.002 15988448

